# Detecting Tumor-Associated Intracranial Hemorrhage Using Proton Magnetic Resonance Spectroscopy

**DOI:** 10.3390/neurolint16060133

**Published:** 2024-12-17

**Authors:** Hye Bin Yoo, Hyeong Hun Lee, Vincent Diong Weng Nga, Yoon Seong Choi, Jeong Hoon Lim

**Affiliations:** 1 Institute for Data Innovation in Science, Seoul National University, Seoul 08826, Republic of Korea; 2 METLiT Inc., Seoul 08513, Republic of Korea; 3 Division of Neurosurgery, Department of Surgery, National University Hospital, Singapore 119228, Singapore; 4 Department of Surgery, Yong Loo Lin School of Medicine, National University of Singapore, Singapore 119074, Singapore; 5 Department of Diagnostic Radiology, Yong Loo Lin School of Medicine, National University of Singapore, Singapore 119074, Singapore; 6 Department of Medicine, Yong Loo Lin School of Medicine, National University of Singapore, Singapore 119074, Singapore

**Keywords:** brain tumor, intracranial hemorrhage, tumoral hemorrhage, proton magnetic resonance spectroscopy

## Abstract

Intracranial hemorrhage associated with primary or metastatic brain tumors is a critical condition that requires urgent intervention, often through open surgery. Nevertheless, surgical interventions may not always be feasible due to two main reasons: (1) extensive hemorrhage can obscure the underlying tumor mass, limiting radiological assessment; and (2) intracranial hemorrhage may occasionally present as the first symptom of a brain tumor without prior knowledge of its existence. The current review of case studies suggests that advanced radiological imaging techniques can improve diagnostic power for tumoral hemorrhage. Adding proton magnetic resonance spectroscopy (1H-MRS), which profiles biochemical composition of mass lesions could be valuable: it provides unique information about tumor states distinct from hemorrhagic lesions bypassing the structural obliteration caused by the hemorrhage. Recent advances in 1H-MRS techniques may enhance the modality’s reliability in clinical practice. This perspective proposes that 1H-MRS can be utilized in clinical settings to enhance diagnostic power in identifying tumors underlying intracranial hemorrhage.

## 1. Introduction

Malignant brain tumors are among the most devastating conditions affecting the human central nervous system. Primary brain tumors like glioblastoma, are rare but fatal, with limited treatment options [1]. Metastatic brain tumors occur in at least 10% of cancer patients and are associated with a poor prognosis [2,3]. Common symptoms of brain tumors include headaches, vomiting, and neurological signs such as seizures and cognitive dysfunction. However, spontaneous intracranial hemorrhage (ICH) can sometimes present as one of the first symptoms of brain neoplasms, including malignant primary or metastatic brain tumors [4,5,6], because brain tumors are prone to malforming angiogenesis and thromboembolism that can easily cause hemorrhage [7,8,9]. Intracranial hemorrhage is a fatal symptom that requires the most urgent and intensive intervention, such as open surgery. The occurrence of hemorrhage prior to the detection of an existing tumor can significantly delay the accurate diagnosis of the underlying tumor and result in a failure to implement the most appropriate clinical measures. Identifying the tumoral origin of spontaneous hemorrhage is critical to prevent dismal consequences [10].

Intracranial hemorrhage impedes the diagnosis of underlying brain tumor by obscuration in radiological images [11,12,13]. Awareness of potential tumor underlying hemorrhage can aid reducing the mortality rate for two main reasons. Earlier detection of tumoral origin can (1) assist precise and on-time interventions, including open surgery, and prevent the further deterioration, especially for brain metastases; and (2) reduce the repeated follow-up procedures to find the main cause of hemorrhage, which is often hidden until the hematoma is completely resolved. Nevertheless, there are major challenges in detecting tumor signs because of limited evaluation on radiological imaging and neurological symptoms that often overlap between ICH and tumor. Based on a review of previous literature, we suggest that proton magnetic resonance spectroscopy (1H-MRS) can provide valuable information for clinical practice targeted at detecting tumor underlying ICH.

This perspective initially examines the neurological implications of tumoral hemorrhage and emphasizes the importance of promptly identifying brain tumors as the underlying cause of ICH. We suggest that integrating 1H-MRS into clinical practice may provide noninvasive and timely detection of tumoral hemorrhage, which may also allow earlier intervention and reduce the need for pre- or intra-operative biopsy and repeated follow-ups after surgery. We further discuss methodological challenges of implementing 1H-MRS in clinical settings and explore potential solutions.

## 2. Materials and Methods

### 2.1. Literature Survey: Criteria for Inclusion and Exclusion

**Figure 1:**
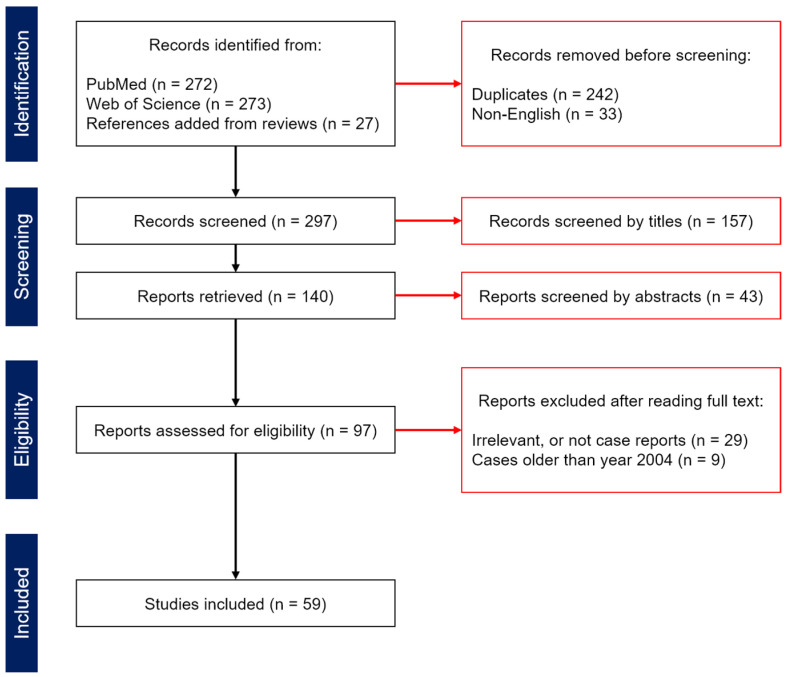
PRISMA plot for selecting case reports (2004–2024) reviewed in the current perspective.

We focus on tumoral hemorrhages directly related to primary or metastatic brain tumors. Our scope excludes cases of hemorrhagic transformation of ischemic stroke without neoplasms in the brain, or hemorrhages occurring due to radiotherapy against tumor masses [14,15], as these cases lack causal links to the diagnosis of brain tumors. We conducted a review of case reports written in English, which were published in 2004–2024 and available on PubMed or Web of Science. Some articles were supplemented from the others’ citations. The keywords used for searching articles in online databases were: (1) (“brain tumor” OR “brain tumour”) AND (“intracranial hemorrhage” OR “intracerebral hemorrhage” OR “intraventricular hemorrhage” OR “intratumoral hemorrhage”) AND (“diagnosis” OR “prognosis” OR “treatment”); in addition to (2) (“brain tumor” OR “brain tumour”) AND (“intracranial hemorrhage” OR “intracerebral hemorrhage” OR “intraventricular hemorrhage” OR “intratumoral hemorrhage”) AND (“magnetic resonance imaging” OR “MR imaging” OR “MRI”) as of 17 July 2024 (Figure 1).

In the process of selecting articles for in-depth review (Figure 1, “Eligibility” step), we aimed to include studies that reported at least one unique case of tumoral hemorrhage. Specifically, we evaluated each paper to determine whether it addressed one or more of the following points: (1) a rare case of tumoral hemorrhage involving an uncommon type of brain tumor or a highly vascularized type of lower-grade tumor; (2) an instance where advanced radiological imaging modalities (i.e., contrast-aided angiography or perfusion-weighted images, diffusion-weighted images, 1H-MRS etc.), excluding positron emission tomography, facilitated accurate diagnosis; (3) a case where the absence of advanced imaging modalities resulted in a missed or delayed diagnosis or intervention; and (4) an example where diagnostic and therapeutic efforts failed despite employing advanced imaging modalities.

### 2.2. Summary of Case Review

Table 1 summarizes the reviewed cases based on patients’ demographics, previously known risk factors for hemorrhage, initial clinical observations, changes in clinical focus for intervention, the state of the underlying tumor, whether patients received immediate open surgery, and their survival status at the time the case was reported. We note that for studies with insufficient data on survival time, particularly for patients shown as “on treatment” in Table 1, we were limited from fully assessing the impact of diagnostic procedures and clinical interventions on patient outcomes. Table 2 presents key insights into the causes, diagnosis, and clinical interventions for tumoral hemorrhage, drawn from recent case reports published over the past five years (2019–2024).

## 3. Results

### 3.1. Overview

We found that tumoral hemorrhage is generally difficult to manage and often necessitates open surgery. Previous reviews have indicated a more frequent incidence of tumoral hemorrhage in malignant neoplasms, such as higher-grade gliomas (e.g., glioblastoma), and brain metastases [5,71], potentially because of the underlying angiogenic factors (e.g., vascular endothelial growth factor) that are more activated in malignant tumor cells [46,52] during their rapid growth [26]. Out of the 62 cases of tumoral hemorrhage we reviewed (Table 1), 39 involved higher-grade brain tumors, including six cases of brain metastases [17,31,37,45,69]. In higher-grade tumors, hemorrhage sometimes presented as the initial and most fatal sign [47]. On the other hand, 23 cases of hemorrhage were associated with lower-grade brain tumors and were potentially linked to significant vascular malformations within the tumor mass [25,57,62]. In some of these cases, patients succumbed to post-intervention hemorrhage [58] or vascular pathologies [55]. Reviews indicate that glioblastoma is more common in adults over the age of 40 [1,72], but there were cases of newborns with tumoral hemorrhage related to congenital glioblastoma [38,60,67].

Although some patients survived tumoral hemorrhage after being diagnosed with benign brain tumors [23,54], missing malignant tumors during the early stages of hemorrhage can lead to critical consequences [11,22,33]. Many cases where tumoral hemorrhage was identified before the discovery of pre-existing brain neoplasms suggest a risk of overlooking the potential connection between hemorrhage and tumors, regardless of the tumor grade. Overall, the manifestation and consequences of tumoral hemorrhage varied, but among the reviewed cases, patients with lower-grade tumors were more likely to survive after intervention (2 out of 23 cases reported death during follow-up) compared to those with higher-grade tumors (13 out of 39 cases reported death during follow-up).

To make a final diagnosis before interventions targeting brain tumors, additional information was often required based on initial observations (Table 1), including biopsies [12,13,19,37]. This aligns with a previous review reporting a median diagnostic delay of 60 days for ICH associated with glioblastoma [6]. Intracranial hemorrhage can delay the accurate diagnosis of brain tumors, especially when monitoring after intervention targeted at ICH is uneventful [27,33]. In certain cases, underlying tumors may arise, with multiple hemorrhagic episodes [53], undergo malignant transformation years after the initial hemorrhagic events [30], or be associated with the healing process following hemorrhagic incidents [42]. These cases suggest that proactive monitoring may be warranted after the initial resolution of hemorrhage.

### 3.2. Common Clinical Practice and Limitations

- Computed tomography

Computed tomography (CT) appears to be the primary imaging tool for visualizing ICH and guiding surgical procedures. Previous studies have shown that emergency screening using CT, such as density attenuation and perihematomal edema volume, can provide clinically relevant information for detecting brain tumor masses [73,74]. In some cases, regular CT can be used to identify tumor mass underlying hemorrhage [18,24,67]. CT angiography may also be useful for distinguishing arteries that feed tumor masses [34,50,51,55], but the signal may be less clear when the mass lesion is not highly vascularized [61]. However, the overt presentation of ICH may hinder detecting tumor masses [11,27], which are less common compared to embolic ischemic stroke or hypertensive hemorrhagic stroke cases [75].

B. Magnetic resonance imaging

Magnetic resonance (MR) imaging provides high-resolution structural information of the gray and white matter integrity and vascular lesions. Advanced MR techniques such as gadolinium contrast-enhanced and diffusion-weighted imaging, enhance the visualization of hemorrhagic tumor masses and offer higher diagnostic power than CT [6,11,21,25,29,33,35,44,46,52,58,63,65,66,68,70] (Table 2). Given the required time and access, imaging modalities that can differentiate blood from tumorous masses should be utilized as early as possible. Advanced MR techniques are less invasive than biopsy and offer higher diagnostic power than CT [35,43,52,58,70]. However, they are only considered when there is clinical evidence and extra time after using first-line imaging tools like CT when ICH is detected [76]. As a result, not all cases of problematic tumor masses are identified using enhanced MR imaging before surgeries targeting hemorrhage [6,11,33,40,44,65,68]. Even if structural MR imaging with contrast enhancement is performed earlier, tumor mass detection may be still challenging due to obscuration by hemorrhage or their location not being aligned with the primary hemorrhagic center [44,65]. There is therefore an unmet need for advanced MR imaging markers that can overcome the limited evaluation on structural T1- and T2-weighted images for early diagnosis. Considering the criticality of treating hemorrhage, the additional imaging marker should be able to identify potential tumor locations quickly (e.g., without requiring different types of scanners or the injection of contrast agents) with higher sensitivity and specificity than structural features. This would enable clinicians to take the necessary risks to perform surgeries targeted at the tumor [41].

C. Tissue biopsy

Histopathological examination offers the most reliable information regarding the characteristics of mass lesions [32,37,49,59], but its availability is inherently restricted. Preoperative biopsy poses critical risks of exacerbating bleeding in the presence of pre-existing hemorrhage and a potential tumor mass [77,78]. Focal sampling of specific regions within a lesion becomes more challenging as the lesion increases in size and exhibits histological heterogeneity. The diagnostic benefits of biopsy may outweigh potential dangers only in situations where less invasive sampling methods, i.e., stereotactic needle biopsy or neuroendoscope [49,52], are feasible. Considering the limitations of relying solely on biopsy as the primary diagnostic tool and the potential challenges of sampling for tumoral hemorrhage, it is advisable to use noninvasive radiological findings whenever possible.

D. Early surgery

Earlier open surgery aimed at removing tumor mass appears to be more advantageous for patients despite its invasiveness. Some studies suggest that less-invasive procedures like neuroendoscope surgery are effective and safe [28,52]. The current review of cases, however, shows that surgical removal of hematoma, hemorrhage, and tumor mass provided more favorable outcomes. Ten out of 33 cases, or 30%, without early open surgery reported death, while five out of 29 cases, or 17%, with early open surgery reported death within the follow-up period (Table 1). An aggressive excision of tumor mass may be required [20] because incomplete removal can result in repeated hemorrhage [64]. Earlier surgery is more effective [16] because the delay may lead to dissemination of tumor cells [36]. Clinicians consider patients’ age before surgery, but there are notable exceptions where open surgery resulted in recovery in a nine-month-old infant [56] and a 94-year-old woman [48]. This creates a dilemma for clinicians on whether to initially choose open surgery for treating apparent ICH—as sometimes, the procedure may not offer significantly greater benefits than conservative approaches [79]—and whether to perform an additional surgery for post-operative symptoms when the follow-up lacks signs of significant progression in the tumor or hemorrhage [39]. It further highlights the significance of utilizing and interpreting radiological evidence to completely exclude the presence of underlying brain tumors before opting for noninvasive treatment for ICH (Table 2).

### 3.3. Clinical Application of 1H-MRS

The major limitations of current clinical practices for detecting tumor masses obscured by hemorrhages can be addressed by employing more accessible and noninvasive MR modalities capable of identifying tumor-specific biomarkers. Proton Magnetic Resonance Spectroscopy (1H-MRS) is a commonly used MR protocol that provides unique information on the distribution of metabolites in the brain, enabling differentiation of tumor masses from normal tissue. We will focus on the biochemical basis of 1H-MRS signals and the clinical procedures for their use in tumor detection.

- Techniques of 1H-MRS in clinical practice

MRS is a noninvasive technique that identifies the biochemical profile of cellular composition in the brain. Specifically, 1H-MRS detects protons (1H) in metabolites that are mobile during the acquisition time and have concentrations above a detectable threshold. While other nuclei, such as carbon (13C) and phosphorus (31P), can be targeted by MRS to provide unique insights into metabolic processes, 1H-MRS remains the most widely used due to its accessibility and compatibility with standard MR imaging equipment (i.e., transmit-receive coils). The latter nuclei require specialized coils for signal acquisition, limiting their use in routine clinical practice.

The most-used acquisition protocols for 1H-MRS are point-resolved spectroscopy (PRESS [80]) and stimulated echo acquisition mode (STEAM [81]), and PRESS is often preferred over STEAM for its superior signal-to-noise ratio (SNR). Because water is the most common source of protons in the brain, a successful acquisition of 1H-MRS must perform adequate suppression of the water signal. Techniques for water suppression, such as chemical shift selective technique (CHESS [82]) or variable power radiofrequency pulses with optimized relaxation delays (VAPOR [83]), reduce the dominant water signal for clearer observation of metabolite signals.

In clinical practice, a single-voxel PRESS scan with water suppression focuses on a specific volume of interest (VOI) in the brain to measure concentrations of important metabolites like choline (*Cho*), N-acetyl aspartate (*NAA*), creatine (*Cr*), and lactate (*Lac*). Since the concentrations of these metabolites can vary based on scan-specific parameters and subject-specific factors such as energy metabolism levels, they are typically expressed as normalized ratios rather than absolute values (e.g., *Cho*/*NAA*, *Cho*/*Cr*).

Higher spatial resolution is necessary to distinguish distinct metabolic profiles in varying types of tissue, such as normal brain tissue, tumor peripheries, and tumor cores. This requires using smaller voxels to acquire spectra [84], but reducing voxel size decreases the SNR, leading to a decline in data quality [85]. Because of this limitation, clinical 1H-MRS typically acquires signals from a few larger VOIs, with average sizes (e.g., 2 × 2 × 2 cm^3^ = 8 cm^3^), significantly larger than those in other MR modalities. The deterioration in data quality when achieving higher spatial resolution (smaller VOI) can be partially mitigated through deep learning (DL)-aided methods, such as denoising techniques [86]. This enables finer spatial resolution tailored to brain lesions, like smaller tumors or cerebral microbleeds, facilitating detailed visualization of brain tissues without significantly increasing scan time.

For more comprehensive spatial coverage, magnetic resonance spectroscopic imaging (MRSI) is an alternative to single-voxel MRS. MRSI reconstructs spatiotemporal (two- or three-dimensional) metabolite information, allowing spectra to be acquired across an entire brain slice or volume and providing spatial advantages over single-voxel MRS. However, routine use of MRSI is hindered by drawbacks such as longer acquisition times and low reproducibility due to variations in reconstruction methods [85].

In summary, the standard clinical application of 1H-MRS involves single-voxel MRS using the PRESS sequence, typically targeting at least two VOIs: a suspected lesion and a normal counterpart. This approach is currently the most practical option for clinical routine, as it balances technical trade-offs in accessibility, signal quality, scan duration, and diagnostic accuracy.

B. Functionality of 1H-MRS in detecting brain tumors

Despite its technical complexity, MRS has been widely used in brain tumor research [87,88]. As of July 17th, 2024, a search on PubMed using keywords (“brain tumor” OR “brain tumour”) AND (“magnetic resonance spectroscopy” OR “MR spectroscopy” OR “MRS”) yielded 713 articles, compared to 285 for dynamic susceptibility contrast MR (“dynamic contrast enhanced” OR “DCE”) and 424 for apparent diffusion coefficient or diffusion-weighted MR (“apparent diffusion coefficient” OR “ADC” OR “diffusion weighted imaging” OR “DWI”), which are two advanced MR modalities commonly used in routine brain tumor analysis. A key diagnostic advantage of 1H-MRS is its ability to distinguish tumor cells from nonneoplastic lesions [89] and differentiate tumor types (primary vs. metastases) or grades [90,91,92,93,94]. Combining 1H-MRS especially with perfusion-weighted MR modalities can enhance diagnostic accuracy for neoplastic pathologies [88,95], significantly benefiting clinical decisions.

Diagnostic 1H-MRS observes metabolites associated with the infiltrative growth of tumorous mass and its metabolic imbalance. As the tumor microenvironment becomes favorable, tumor cells aggressively acquire resources to sustain proliferation, leading to increased hypoxia, angiogenesis, and invasion of surrounding tissues by degrading basement membranes [7]. This process produces contrasting characteristics of tumor mass compared to the normal, which are abnormal energy metabolism and reduced cell integrity due to increased necrosis, and elevated membrane turnover in tumor-adjacent regions [96,97]. Key findings in the tumorous tissue include (1) elevated *Lac*: abnormal anaerobic metabolism increases concurrently with the concentration of *Lac* that is absent in normal spectra; (2) reduced *NAA*: more normal cells in the mass are destroyed, reflected by lower levels of neuronal integrity probed by *NAA* concentration; and (3) elevated *Cho*: as tumor cells rapidly proliferate and infiltrate the neighbor tissues, the rate of cell membrane turnover rises and the concentration of *Cho*, a component of cell membranes, also increases [98].

**Figure 2:**
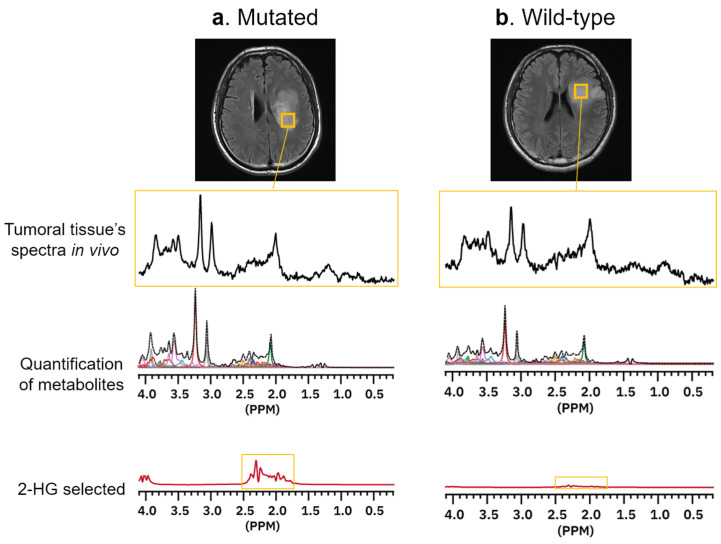
Comparison of 2-hydroxyglutarate (*2-HG*) concentration between two cases that have either (**a**) IDH1 mutation or (**b**) wild-type. Tumoral tissues visually inspected are sampled using structural MR (highlighted by yellow boxes), and the metabolite concentrations are quantified using AI-aided processing of MR spectra (MRS Analytics) developed by METLiT Inc.

A normalized *Cho*/*NAA* ratio is a particularly informative marker for brain tumor diagnosis, signaling increased membrane turnover alongside reduced cell viability [92,96,99,100]. Additionally, 1H-MRS has unique sensitivity to 2-hydroxyglutarate (*2-HG*), a metabolite elevated exclusively in tumor cells with the oncogenic isocitrate dehydrogenase (IDH1) mutation [101,102], frequently found in gliomas [103] and secondary glioblastomas [104]. Since IDH1 mutation is associated with prolonged survival and better response to chemotherapy [105,106], 1H-MRS is highly informative for these subtypes of brain tumors [107,108]. Figure 2 demonstrates how 1H-MRS identifies tumor mass that is positive for the IDH1 mutation by quantifying *2-HG*.

For clinical interpretation of MRS signals, metabolite markers are compared across multiple regions in the brain (e.g., potential tumor mass vs. normal region). Consequently, MRS requires a predefined spatial framework for data acquisition and is generally conducted after structural MR and a visual inspection of structural abnormalities where applicable. A typical set of regions of interest in clinical routine for cases of suspected brain tumor would include (1) suspected tumor mass and (2) normal tissue often positioned contralateral to the suspected mass lesion.

C. Implementation of 1H-MRS in detecting tumoral origin of hemorrhage

Currently, 1H-MRS is a supplementary choice for identifying brain tumors because its biochemical markers can overlap across different pathologies that induce abnormal anaerobic metabolism or necrosis, such as infections, inflammatory diseases, neurodegenerative disorders, and stroke [87,96]. Nevertheless, 1H-MRS can add its clinical utility to structural MR when the ICH is already discovered. The conditional probability of ICH increases given an underlying tumorous mass that demonstrates hypervascularization, overactive angiogenesis, and infiltration into neighboring tissues and blood vessels [7,109,110]. Thus, the incidence of ICH can narrow down the diagnostic ranges and add spatial specificity for employing MRS to scan for suspected tumors. Despite the lack of contrast-aided high-resolution MR, 1H-MRS can detect increase in *Cho*/*NAA* or *Cho*/*Cr* indicating higher membrane turnover related to cell proliferation, which is a marker of a tumor mass independent of hemorrhagic damage [96]. In summary, 1H-MRS can be considered a noninvasive “virtual biopsy” that bypasses visual obstructions caused by hemorrhage and probes the metabolic state from proliferation to necrosis in hemorrhagic tissues.

Tumoral hemorrhage is assessed through multiple aspects in practice. First, patient clinical records provide clues about the causes of hemorrhage. Patients without apparent physical injuries or common risk factors for hemorrhage (e.g., hypertension) should be evaluated for possible primary brain tumors, while those with a history of cancer should be assessed for metastatic brain tumors. Structural MRI plays a key role in identifying the hemorrhagic center and assessing image intensity heterogeneity, which may indicate an underlying tumor mass. Single-voxel 1H-MRS can be applied to at least three VOIs: (1) the center of the hemorrhagic lesion, (2) the perilesional area (usually avoiding regions clearly filled with fluid), and (3) normal tissue located distant from the suspected mass lesion (usually contralateral to the lesion, but if not applicable, areas that are not affected by the hemorrhagic event).

**Figure 3:**
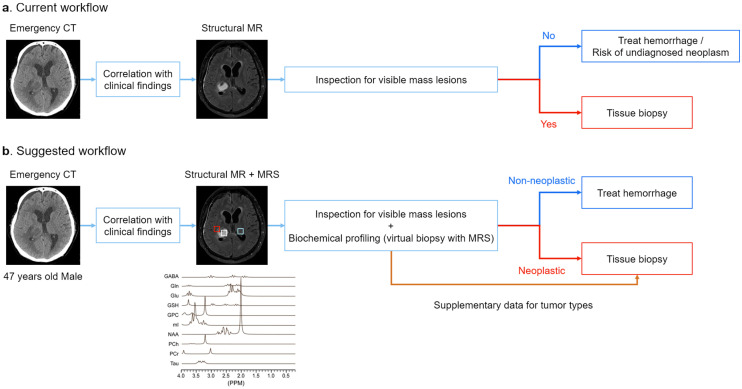
Suggested workflow compared to the current approach for utilizing MRS to detect tumor masses obscured by hemorrhage. (**a**) In current clinical practice, when emergency CT scans reveal intracerebral hemorrhagic lesions, structural MRI is often considered as a follow-up after clinical correlation. However, treatment decisions may rely heavily on imaging findings, potentially overlooking underlying neoplastic conditions. (**b**) By integrating structural MRI with MRS for cases of suspected hemorrhagic lesions observed on CT, clinicians can obtain both structural details and a biochemical profile of the lesion. We propose three volumes of interest: (1) normal tissue often positioned contralateral to the lesion (cyan), (2) suspected tumor mass (white), and (3) perilesional area (red). If the suspected lesion exhibits a spectral profile consistent with that of neoplasms and significantly differs from the profiles of normal and perilesional regions, clinicians may proceed with interventions specifically targeting the tumor mass. This suggested workflow may enhance diagnostic sensitivity and accuracy, especially when the neoplastic mass is small or obscured by hemorrhage. MRS can also provide information regarding the tumor type, which could inform biopsy strategies and further clinical decisions.

We propose that patients presenting with ICH on CT, particularly those without common risk factors for ICH (e.g., hypertension or significant physical trauma) or those with a history of cancer (raising the possibility of metastatic brain tumors), may benefit more from clinical use of 1H-MRS. The contraindications and risks associated with 1H-MRS are like those of other MR modalities. In resource-limited settings, prioritizing 1H-MRS for patients with ICH and a clinical suspicion of neoplasm can help optimize diagnostic and treatment strategies. Figure 3 presents a suggested workflow compared to the current approach for utilizing MRS to detect tumor masses obscured by hemorrhage.

Despite the potential utility of 1H-MRS targeting tumor underlying hemorrhage, there are some technical difficulties to overcome for its reliable use. One concern is the susceptibility artifact that causes signal loss, or spectral distortion in hemorrhagic regions, caused by blood products [111]. These distortions are not limited to hemorrhagic areas but also commonly occur in surrounding regions, including non-tumoral areas such as necrotic tissue (due to necrosis or apoptosis) and edematous regions. When tumoral mass overlaps with hemorrhagic regions, the resulting metabolomic information may become mixed unless sufficient spatial resolution is achieved to differentiate them, a challenge that may be partially addressed by employing MRSI at the cost of longer scan times. Another challenging aspect of MR imaging, particularly in brain tumor patients, is frequent patient movement. During MRS acquisition, the movement effect can significantly degrade data quality, potentially compromising the accuracy required for metabolite quantification. Although excessive movement may necessitate repeated scans, some recent advancements for refining 1H-MRS spectra can improve signal reliability, and they may enable more routine use of 1H-MRS in regions prone to susceptibility artifacts. The next section discusses recent advancements in MRS processing techniques that aim to overcome technological challenges in detecting tumoral hemorrhages.

### 3.4. Enhancing Data Quality of 1H-MRS

- Recent technological advances in MRS analysis

One of the major advances in MRS is related to data processing rather than optimizing its hardware. Specifically, the DL-aided approach has improved the precision of metabolites quantification [112]. This approach is more practical and feasible compared to improving the quality of the original MRS signal by increasing the magnitude of the magnetic field (from 3T to 7T), optimizing scan parameters, or adding MR modalities to accurately segment across gray matter, white matter, and cerebrospinal fluid.

The metabolite concentration in the MRS signal is quantified by fitting an algorithm, such as nonlinear least squares fitting (NLSF), which estimates the MR spectra of given metabolites at a specific concentration level. Fitting algorithms typically used in practice include LCModel [113] or QUEST [114]. They decompose complex spectra into individual metabolite signals in the frequency or time domain, accounting for signal overlapping and baseline distortions. The reliability of quantitative results can be assessed by the Cramér–Rao Lower Bound (CRLB), which expresses the uncertainty of each metabolite concentration as a percentage [113]; lower CRLB values indicate higher level of confidence in the metabolite concentrations estimated by NLSF, or the higher “precision”.

Recent DL-aided techniques have been focused on not only improving performance compared to traditional NLSF-based fitting algorithms, but also overcoming the technical limitations presented by the CRLB value. A Bayesian deep neural network model incorporating an approximated variational inference principle with Monte Carlo sampling has been developed [115,116,117]. This model offers statistical uncertainty that reflects levels of both accuracy and precision and achieves a lower absolute quantification error rate compared to the conventional NLSF method by accounting for systemic errors in the model and noise in the input. This capability enables robust metabolite quantification with lower data quality (e.g., lower SNR) depending on the diversity and quantity of training data. This advancement can potentially reduce the scan times required for the standard of spectral signal quality in MRS.

B. Setting scan parameters

Configuration of scan parameters alters the types of metabolites that can be detected or quantified by 1H-MRS. Optimal setting of scan parameters is therefore critical for maximizing diagnostic capability for tumors underlying hemorrhage [118,119]. One key parameter is echo time (TE). Short-TE MRS maximizes signal yields but also amplifies signals from lipids and macromolecules, leading to spectral overlaps with metabolites of interest, which necessitates post-processing for precise separation [120]. Long-TE MRS focuses on some tumor-specific metabolites like *Lac* [121] or *2-HG* [122], but limits the observation of complex metabolite changes within the tumor.

To overcome these issues, some recently developed methods that employ convolutional neural network-based DL approaches for quantifying metabolites in MR spectra analysis. Most of these methods aim to extract the unique features of individual metabolite spectra using short TE only [86,123,124]. Other DL-aided approaches attempt to quantify metabolites from edited spectra or to automatically adjust frequency and phase distortions as part of the post-processing to enhance spectral signal quality [125,126]. Developing and integrating appropriate neural network architectures trained for each MRS scan protocol may lead to further methodological advancements to improve the quantitative performance for detecting changes in specific metabolites within tumors and other heterogeneous in vivo masses.

C. Spectral distortions

As mentioned in the previous section, spectral distortion in hemorrhagic regions is an important concern regarding data quality in 1H-MRS. Blood products in the hemorrhagic center create paramagnetic deoxyhemoglobin, which disrupts the local magnetic field and causes field inhomogeneity, leading to lower SNR and spectral distortions [111]. This results in lower-quality signals from the hemorrhagic region [127], posing technological challenges in detecting tumor markers within larger hemorrhagic centers.

The quality of MR spectra in tumoral regions is also lower compared to nonneoplastic areas [128]. Complex biological processes, i.e., cell proliferation, apoptosis, and necrosis resulting from various genetic mutations and biological changes in tumor cells relate to lower quality of the MR signal [96]. Tumor-related processes also lead to rapid shifts in brain metabolic profiles such as an increase in lipid production [129]. Consequently, magnetic field homogeneity in tumor masses is poorer, directly impacting the quality of MR spectra acquisition, resulting in lower SNR and broader spectral linewidth [85].

Considering the specific characteristics of spectral signals in brain tumor regions with hemorrhage, recent advances in DL-aided preprocessing methods can enhance signal quality acquired from hemorrhagic centers. These methods are useful for determining whether the acquired MR spectra are suitable for analysis and if the data distortion is within an acceptable range. They can also be utilized for restoring lower-quality data to improve the accuracy of quantifying metabolites in the tumor. For instance, DL-aided methods can identify spurious echoes [130], suppress lipid signals [131], assess the validity of quantification based on the extent of signal distortion [132,133], and attempt to recover signals [134]. Some methods provide uncertainty information for machine-driven quantification results to measure reliability [115,116]. This information can guide users in assessing the risks of using unstable quantification results in radiological interpretation or for research purposes. In summary, DL-aided preprocessing techniques can be employed for improving the quality of MR spectral data from hemorrhagic brain tumor regions, evaluating signal distortion and the validity of metabolites’ quantification, and restoring lower quality data for the more effective use of MRS in clinical practice and research.

D. Suggestions for future research directions

Future methodological studies regarding 1H-MRS should aim to develop robust solutions for addressing limitations that reduce its diagnostic accuracy in clinical practice. Two major issues are the lower spatial resolution and signal quality of MRS, which can be particularly problematic for smaller and irregularly shaped tissues and nonhomogeneous structures with a significant amount of fluid. Improving spatial resolution could be achieved by increasing the number of VOIs covering the area of interest or mapping VOIs with customized boundary shapes. These techniques can enhance the accuracy of diagnoses and facilitate the virtual shaping of tumor masses to aid surgical removal. In practical applications of MRS for tumoral hemorrhage, it is essential to ensure spectral quality required for quantification despite the presence of hemorrhage and other nonhomogeneous lesions. For precise quantification of metabolites in MRS targeting brain tumor regions, where the concentration of most metabolites is significantly degraded compared to normal structures, more strict cutoff criteria for spectral quality should be applied. Current advancements in DL-aided methods for spectral data processing may help tackle challenges in recovering and improving MRS signal quality. The use of DL-aided methods for 1H-MRS data processing is growing, with mounting evidence for the modality’s potential in addressing clinically challenging pathologies like brain tumors. [108,135,136]. Future MRS research should aim for the more robust data collection in multiple hospitals to differentiate between primary or metastatic brain tumors and blood masses or ischemic lesions in patients with ICH.

Increasing the accessibility of 1H-MRS would be essential for its wider adoption in hospitals, particularly those with limited access to advanced MR modalities. Recent techniques for spectral data processing require less scan time (around an additional ten minutes for two or three VOIs in single-voxel MRS) for 1H-MRS acquisition, thereby aiding its employment. The application of DL-aided techniques further reduces reliance on the dedicated MRS specialists for data interpretation to enhance the modality’s practicality. These advancements in 1H-MRS software can enhance its utility in routine clinical practice.

## 4. Discussion

Hemorrhagic events, particularly in patients without known risk factors, should be promptly assessed for the possibility of an underlying brain tumor. Earlier surgical intervention to remove suspected hemorrhagic tumors probably leads to better outcomes, but it is essential to use noninvasive clinical imaging like advanced MR imaging for the more accurate diagnosis before resorting to surgery or biopsies to minimize unnecessary invasive procedures. Among various advanced MR modalities, 1H-MRS provides insights into biochemical profiles specific to tumor tissues, even when hemorrhage obscures visual inspection. Recent advancements in deep learning-aided methods for processing 1H-MRS data have improved the accuracy for quantifying brain metabolites, which may further enhance its diagnostic utility for brain tumors in clinical practice. Future 1H-MRS research should focus on reinforcing its clinical robustness by analyzing datasets from diverse patient pools across multiple healthcare sites and incorporating recently developed techniques to increase spatial resolution.

## Figures and Tables

**Table 1:** An alphabetically sorted list of reviewed cases of years 2004–2024 (59 studies, 62 cases). Risk factor represents any significant medical records that directly relate to the present tumoral hemorrhage. Initial observation refers to the apparent medical condition for which clinicians originally sought resolution. Change in focus may have happened during or after primary treatment based on the initial observation. The grade of underlying tumors refers to WHO grade I or II (lower) or III or IV (higher). Open surgery refers to the invasiveness of the immediate first-step treatment procedures.

Cases	Patient	Risk Factor	Initial Observation	Change in Focus (Cause)	Underlying Tumor	Grade	Open Surgery	Survival
Abuzayed, Khreisat (2014) [16]	F 24	N/S	Mass lesion	No	PNET	Higher	No	On treatment
Akasaki, Tsutsumi (2023) [17]	F 78	Non-brain tumor	CCM	Yes (MR)	Brain metastases	Higher	No	On treatment
Bosnjak, Derham (2005) [18]	F 44	N/S	Mass lesion	No	Meningioma	Lower	No	Recovered
	M 74	Stroke and diabetes	Stroke	Yes (CT)	Meningioma	Lower	No	Recovered
Bruscella, Alfieri (2021) [19]	M 17	N/A	ICH	Yes (Surgery)	Sarcoma	Higher	No	On treatment
Burkhardt, Kockro (2011) [13]	M 16	N/S	ICH	Yes (Biopsy)	PNET	Higher	Yes	On treatment
Carrasco, Pascual (2010) [20]	M 71	Hypertension and chronic atrial fibrillation	Mass lesion	No	Subependymoma	Lower	Yes	Recovered
Choi, Park (2013) [21]	F 69	Hypertension	ICH	Yes (Enhanced MR)	Astrocytoma	Higher	No	On treatment
Datta, Datta (2006) [22]	M 7	N/S	ICH	Yes (MR)	GBM	Higher	No	Passed, a few days
De Almeida, Petteys (2009) [23]	F 66	Meningioma	Meningioma	No	Meningioma	Lower	Yes	Recovered
De Sousa, Rego (2022) [24]	F 31	N/S	Hearing loss	Yes (CT)	Schwannoma	Lower	No	Recovered
Donofrio, Gagliardi (2020) [25]	M 9	N/S	Mass lesion	No	Astrocytoma	Lower	No	Recovered
Duan, Kitazawa (2016) [26]	F 71	Hypertension and aneurysm	Mass lesion	No	Sarcoma	Higher	Yes	Recovered
Eom, Kim (2020) [27]	M 40	Hypertensive ICH	ICH	Yes (Angiography)	SFT/HPC	Higher	Yes	Recovered
Fuchinoue, Uchino (2022) [28]	M 81	Brain tumor	ITH	No	Subependymoma	Lower	No	Recovered
Grimm, Deangelis (2007) [29]	M 80	N/A	Mass lesion	Yes (Enhanced MR)	GBM	Higher	No	Not shown
Han, Park (2014) [30]	M 23	ICH	ICH	Yes (MR)	Ependymoma	Higher	Yes	On treatment
Hanada, Oyoshi (2010) [31]	M 69	N/S	Mass lesion	No	Brain metastases	Higher	No	Passed, 4 months
	M 37	Non-brain tumor	Mass lesion	No	Brain metastases	Higher	No	Passed, 8 months
Hu, Zhang (2018) [32]	M 58	N/A	Mass lesion	No	Meningioma	Higher	Yes	N/A
Inamasu, Kuramae (2009) [33]	M 58	Hypertension and diabetes	ICH	Yes (Enhanced MR)	GBM	Higher	No	Passed, 3 months
Inamasu, Nakamura (2005) [34]	F 42	N/S	ITH	No	GBM	Higher	No	Passed, 90 days
	F 68	N/S	ICH	Yes (Angiography)	GBM	Higher	No	On treatment
Ito, Nakajima (2015) [35]	F 78	Brain tumor	Mass lesion	Yes (MR)	Meningioma	Higher	No	On treatment
Iwamoto, Murai (2014) [36]	M 61	N/A	Mass lesion	No	Ependymoma	Higher	No	N/A
Jang, Kim (2015) [37]	M 51	Non-brain tumor	ICH	Yes (Biopsy)	Brain metastases	Higher	Yes	Passed, 8 days
Joseph, O’neill (2017) [6]	F 21	N/A	ICH	Yes (Enhanced MR)	GBM	Higher	Yes	On treatment
Junior, Abreu (2022) [38]	Newborn	N/S	Mass lesion	No	GBM	Higher	Yes	On treatment
Kawashima, Hasegawa (2021) [39]	M 64	N/S	Schwannoma	No	Schwannoma	Lower	No	Recovered
Kim, Jung (2008) [40]	F 49	N/S	Mass lesion	No	Lymphoma	Higher	No	On treatment
Kim, Jung (2017) [41]	F 10	N/A	ICH	Yes (MR)	Astrocytoma	Lower	Yes	Recovered
Kim, Lee (2008) [42]	M 64	ICH	Mass lesion	No	GBM	Higher	Yes	On treatment
Li, Wang (2013) [11]	M 61	Hypertension and diabetes	Cerebral contusion	Yes (Enhanced MR)	GBM	Higher	No	Passed, N/A
Liebelt, Boghani (2015) [12]	F 66	Hypertension and non-brain tumor	Mass lesion	Yes (Biopsy)	GBM	Higher	Yes	Not shown
Ma, Jia (2019) [43]	M 26	N/S	Mass lesion	No	Gangliocytoma	Lower	Yes	Recovered
Marfia, Pirola (2018) [44]	M 36	N/A	ICH	Yes (MR)	Neurocytoma	Lower	Yes	Recovered
Matsuo, Amano (2019) [45]	F 61	Non-brain tumor	Mass lesion	No	Brain metastases	Higher	Yes	Passed, 3 weeks
Matsuyama, Ichikawa (2014) [46]	M 67	N/A	Neuritis	Yes (Enhanced MR)	Lymphoma	Higher	No	Passed, 3 years
Menekse, Gezercan (2015) [47]	F 8	N/S	Mass lesion	No	Medulloblastoma	Higher	Yes	Passed, short
Miyashita, Nambu (2023) [48]	F 94	Brain tumor	Mass lesion	No	Meningioma	Lower	Yes	Recovered
Miyazaki, Tsuji (2019) [49]	F 26	N/A	Mass lesion	No	Glioma	Higher	No	Passed, 3 weeks
Miyazawa, Hirose (2007) [50]	M 33	N/A	ICH	Yes (Angiography)	Ependymoma/ GBM	Higher	No	Recovered
Moon, Cha (2019) [51]	F 35	N/A	Mass lesion	No	Meningioma	Lower	No	Recovered
Muroya, Suzuki (2023) [52]	F 75	Brain tumor	Tumor lesion	No	Lymphoma	Higher	No	Recovered
Pagano, Novegno (2019) [53]	M 22	Mass lesion with hemorrhage	Mass lesion	No	Astrocytoma	Lower	No	Recovered
Parenrengi, Aji (2020) [54]	F 14	Brain tumor	Mass lesion	No	Astrocytoma	Lower	Yes	Recovered
Pressman, Penn (2020) [55]	F 56	Aneurysm and asystole	Mass lesion	Yes (Angiography)	Meningioma	Lower	No	Passed, 2 weeks
Ramdurg, Maitra (2016) [56]	9 months	N/S	Mass lesion	No	Astrocytoma	Lower	Yes	Recovered
Ritz, Roser (2005) [57]	M 47	N/A	Mass lesion	No	Neurocytoma	Lower	Yes	Recovered
Sachani, Dhande (2024) [58]	F 35	Chronic headache	Mass lesion	No	Neurocytoma	Lower	Yes	Passed, 40 days
Sangatsuda, Hata (2018) [59]	M 80	ICH	Mass lesion	No	Glioma	Higher	Yes	Passed, 28 months
Seker, Ozek (2006) [60]	Newborn	Cranial abnormalities	Mass lesion	No	GBM	Higher	Yes	On treatment
Seki, Kamide (2016) [61]	F 64	N/A	ICH	Yes (Angiography)	Hemangio- pericytoma	Higher	Yes	On treatment
Shibao, Kimura (2012) [62]	M 29	N/A	Mass lesion	No	Astrocytoma	Lower	Yes	Recovered
Singla, Aggarwal (2016) [63]	M 60	Hypertension	Aneurysm	Yes (Enhanced MR)	GBM	Higher	Yes	Not shown
Sorimachi, Sasaki (2008) [64]	F 37	N/A	Mass lesion	No	Chondrosarcoma	Lower	Yes	Recovered
Soto, Lyon (2018) [65]	F 52	Lupus and diabetes	ICH	Yes (MR)	GBM	Higher	No	On treatment
Takamine, Yamamuro (2019) [66]	F 11	N/S	ICH	Yes (Enhanced MR)	Astrocytoma	Lower	No	Recovered
Thankamony, Harlow (2007) [67]	Newborn	Abnormal fetal movements	ICH	Yes (CT)	GBM	Higher	No	Passed, 2 days
Tseng, Lin (2012) [68]	M 72	N/S	ICH	Yes (Enhanced MR)	GBM	Higher	No	On treatment
Yamashita, Fukuda (2011) [69]	F 57	Non-brain tumor	ICH	Yes (MR)	Brain metastases	Higher	No	On treatment
Yindeedej, Rojnueangnit (2024) [70]	M 16	N/S	Mass lesion	No	Astrocytoma	Lower	Yes	Recovered

Abbreviations: ICH = Intracranial hemorrhage, SFT/HPC = Solitary Fibrous Tumor/Hemangiopericytoma, MR = Magnetic resonance (imaging), CT = Computerized tomography, ITH = Intratumoral hemorrhage, GBM = Glioblastoma multiforme, CCM = Cerebral cavernous malformations, PNET = Primitive neuroectodermal tumor, N/S = Nothing significant, N/A = Not available.

**Table 2:** Summary of key findings and insights on tumoral hemorrhage, derived from a review of 20 studies (20 clinical cases) published between 2019 and 2024.

Key Findings	Insights
Causes of tumoral hemorrhage	- Caused by rare and malignant brain tumors (e.g., intracerebral nerve sheath tumors [19], metastatic tumors [45], meningiomas [51]) - Triggered by minor injuries [17], clinical interventions like stereotactic radiosurgery [48], or administration of anticoagulants [39] - Tumors may remain undiagnosed for extended periods, leading to fatal outcomes, especially in children or benign cases due to the sparsity of medical records [24,49]
Diagnostic challenges	- Brain tumors (e.g., pilocytic astrocytoma, glioblastoma, subependymal giant cell astrocytoma) can be asymptomatic or misdiagnosed [25,38,43,70] - Symptoms in pediatric cases may be vague or absent, delaying diagnosis [25] - Advanced imaging techniques (e.g., MR scans with perfusion/diffusion-weighted imaging, contrast-enhanced MR, CT angiography) are important for accurate detection [52,66,70]
Treatment considerations	- Less-invasive procedures (e.g., neuroendoscopic surgery) can treat some tumors (e.g., lateral ventricular subependymoma) [28] - Tumoral hemorrhages may persist after surgery due to neoplastic angiogenesis [58] - Postoperative hemorrhage can be fatal if not managed carefully (e.g., neurocytoma resection) [58]
Complications from medical treatment	- Anticoagulants [48] and serotonin-modulating therapy increase the risk of hemorrhage, particularly in meningioma patients [55] - Regular monitoring and follow-up scans are crucial for early diagnosis and better prognosis [39]
Long-term effects and complications	- Tumors (e.g., pilocytic astrocytoma) may be diagnosed many years after a hemorrhagic event with potential for malignant transformation [53]

## Data Availability

Data sharing is not applicable because no new data were created for the purpose of this study.
